# Development and immunogenicity of a novel subunit vaccine candidate against avian metapneumovirus

**DOI:** 10.3389/fvets.2026.1907159

**Published:** 2026-08-13

**Authors:** Urzhan Omarbekova, Aspen Abutalip, Yergali Moldakhanov, Madina Alexyuk, Assylbek Mussoyev, Indira Mussayeva, Andrey Bogoyavlenskiy, Pavel Alexyuk

**Affiliations:** 1 Department of Biological Safety, Faculty of Veterinary and Zooengineering, Kazakh National Agrarian Research University, Almaty, Kazakhstan; 2 Laboratory of Antiviral Protection, Research and Production Center of Microbiology and Virology, Almaty, Kazakhstan

**Keywords:** aMPV subtype B, avian metapneumovirus, cytokines, Kazakhstan, poultry, subunit vaccine, virus neutralization

## Abstract

**Objectives:**

aMPV subtype B remains one of the major respiratory pathogens affecting commercial poultry worldwide despite the widespread implementation of vaccination programs. In the present study, epidemiological surveillance, virus isolation, and immunogenic evaluation of a newly developed subunit vaccine against aMPV subtype B were conducted.

**Methods:**

Clinical samples, representing 115 chicken flocks from 16 commercial poultry farms located in three regions of Kazakhstan, were screened for aMPV subtype B using multiplex real-time RT-PCR. Approximately 20% of the samples collected during the spring season tested positive for aMPV, with subtype B predominating among the detected strains. Phylogenetic analysis revealed that the identified viruses clustered within subtype B and were closely related to vaccine-like strains while forming a distinct monophyletic group.

For virus isolation, field samples that tested negative for IBV, NDV, ILTV, and *Mycoplasma gallisepticum* were propagated in Vero cells. Cytopathic effects were observed after 3–4 passages, and one isolate, designated Ck/B/Kaz/256/25, was successfully recovered from a laying hen. The isolate was subsequently confirmed by electron microscopy, gel electrophoresis, and was used for the development of a subunit vaccine.

**Results:**

The immunogenicity of the vaccine was evaluated in chickens following a prime–boost immunization regimen and compared with that of a commercial live vaccine. The subunit vaccine induced 100% seroconversion by day 14 post-vaccination and elicited significantly higher IgG and virus-neutralizing antibody titers than the live vaccine. Neutralizing antibody titers were 8-fold higher after primary immunization and 16-fold higher after booster immunization compared with those in the live vaccine group. Furthermore, the subunit vaccine significantly enhanced the expression of IFN-γ and IL-4, indicating activation of both Th1- and Th2-mediated immune responses.

**Conclusion:**

Overall, these findings demonstrate that the developed subunit vaccine induces robust and durable humoral and cellular immune responses and represents a promising candidate for the prevention and control of aMPV subtype B infection in poultry.

## Introduction

aMPV subtype B infection continues to represent a major challenge for the poultry industry worldwide and is considered one of the most important viral respiratory diseases of commercial birds despite the extensive use of vaccination and biosecurity programs (1, 2). The pathogen belongs to the genus *Metapneumovirus* within the family *Paramyxoviridae* and primarily targets epithelial tissues of the upper respiratory tract (3). Infection can lead to turkey rhinotracheitis, swollen head syndrome in chickens, reduced egg production, growth retardation, and increased vulnerability to opportunistic infections. In addition to direct economic losses caused by mortality and decreased productivity, aMPV subtype B outbreaks are associated with substantial indirect costs related to secondary bacterial infections, deterioration of feed efficiency, impaired flock uniformity, and increased application of antimicrobial agents (4).

The epidemiological relevance of aMPV subtype B is largely associated with the extensive genetic heterogeneity of circulating strains. Currently, four major subtypes (A–D) together with newly emerging variants have been described, exhibiting considerable molecular and antigenic differences (5). This diversity complicates the induction of broad cross-protective immunity and favors persistent viral circulation in high-density poultry production systems. Furthermore, intensive housing conditions facilitate rapid transmission of the virus and contribute to the occurrence of mixed respiratory infections involving pathogens such as Avibacterium paragallinarum, Ornithobacterium rhinotracheale, and infectious bronchitis virus, which may aggravate clinical manifestations and further reduce production performance (6).

At present, prophylaxis against aMPV subtype B mainly relies on the application of live attenuated and inactivated vaccines; however, both approaches possess significant drawbacks (7, 8). Live vaccines may provoke post-vaccination respiratory disturbances, show limited efficacy against antigenically distinct field strains, and potentially undergo reversion to virulence after circulation in poultry populations. Although inactivated vaccines are generally safer, they often induce relatively weak mucosal and cellular immune responses and therefore require effective adjuvants and multiple booster administrations to maintain long-term protection. Moreover, vaccine-induced immunity under commercial production conditions is frequently insufficiently durable.

Recent progress in molecular biotechnology and vaccine engineering has promoted increasing interest in the development of recombinant and subunit vaccine platforms against aMPV subtype B (9, 10). Unlike traditional vaccines, subunit preparations are based on purified immunogenic viral proteins, predominantly the surface glycoproteins F and G, which are essential for viral attachment, membrane fusion, and stimulation of neutralizing antibody responses. The use of selected viral antigens significantly improves biosafety by eliminating infectious viral particles and also enables modification of antigen composition according to the currently circulating strains.

Considerable attention has recently been focused on combining subunit antigens with innovative adjuvant and delivery technologies (11, 12). Various nanostructured systems, including lipid nanoparticles, saponin-containing immunostimulating complexes, polymer-based nanoparticles, and virus-like particles, have demonstrated promising immunomodulatory properties by enhancing antigen stability, facilitating antigen uptake, and promoting both humoral and cellular immunity. The combination of safety, manufacturing scalability, batch-to-batch consistency, and support for DIVA (Differentiating Infected from Vaccinated Animals) programs has made these platforms attractive solutions for modern veterinary vaccine design. (13, 14).

Consequently, the development of subunit vaccines against avian metapneumovirus may provide an effective alternative to conventional vaccination strategies and contribute to improved control of aMPV subtype B infection in intensive poultry production.

## Materials and methods

### Materials

*Vaccine* Live attenuated vaccine HIPRAVIAR SHS (Laboratorios HIPRA, Spain) for the prevention of Turkey rhinotracheitis (TRT) and Swollen Head Syndrome (SHS) associated with avian metapneumovirus (aMPV) infection.

The vaccine contains a live attenuated avian metapneumovirus (Turkey rhinotracheitis virus), strain 1062, with a minimum potency of 10^2.4^ TCID_50_ per dose. The product is supplied as a freeze-dried tablet for reconstitution prior to administration.

HIPRAVIAR SHS is intended for the immunization of turkeys, broiler chickens, layer chickens, and breeder chickens to protect against the clinical manifestations of turkey rhinotracheitis and swollen head syndrome.

The recommended vaccination dose is one dose per bird. Following reconstitution, the vaccine may be administered by the ocular, intranasal, oral, or spray routes. For ocular or intranasal vaccination, a single drop (0.03 mL) of the reconstituted vaccine is instilled into one eye or one nostril of each bird.

*Chickens* One-week-old commercial chickens were obtained from commercial flocks (Almaty, Kazakhstan) and housed in positive-pressure isolators under controlled environmental conditions with free access to feed and water. All animal procedures were approved by the institutional bioethics committee and performed in accordance with international guidelines for animal welfare.

### Clinical sample collection

Clinical samples were collected during 2024–2025 as part of surveillance studies investigating the circulation of aMPV subtype B in commercial poultry farms located in different regions of Kazakhstan. A total of 115 chicken flocks were included in the study, comprising 15 broiler flocks, 87 broiler breeder flocks, and 13 layer flocks. Birds exhibited respiratory signs of varying severity, including nasal discharge, swollen infraorbital sinuses, tracheal rales, and reduced egg production, although some flocks remained clinically asymptomatic.

From each flock, oropharyngeal swabs were collected from six birds and pooled at a ratio of two swabs per sample (15). Material obtained from the oropharynx and nasal turbinates was suspended in minimal essential medium supplemented with gentamicin. Following centrifugation at 1000 × g for 10 min, clarified supernatants were used for molecular and virus isolation.

### Methods

#### RNA extraction and RT-qPCR detection of aMPV subtype B

Total RNA was extracted from samples using the RNeasy Mini Kit (Qiagen, Hilden, Germany) according to the manufacturer's instructions. RNA concentration and purity were assessed using a NanoDrop spectrophotometer, and only samples with an A260/A280 ratio of 1.8–2.0 were used for cDNA synthesis.

Detection of aMPV subtype B RNA was performed by real-time reverse transcription PCR (RT-qPCR) using specific primers and TaqMan probes targeting conserved regions of the viral genome (16, 17). The reaction mixture (25 μL) contained 10 μL of extracted RNA, 12.5 μL of QuantiTect Probe RT-PCR Master Mix, 0.25 μL of QuantiTect RT Mix enzyme solution, and primers and probes at optimized concentrations.

Amplification was performed using a QuantStudio 5 system (ThermoScientific, USA) under the following conditions: reverse transcription at 50 °C for 30 min, initial denaturation at 95 °C for 15 min, followed by 45 cycles of 94 °C for 15 s and 55 °C for 60 s. Samples positive for aMPV subtype B RNA were subsequently used for virus isolation.

#### Cell culture and virus propagation

Virus isolation and propagation were performed in Vero E6 cells maintained in Dulbecco's Modified Eagle Medium (DMEM; Sigma, China) supplemented with 10% fetal bovine serum (FBS), penicillin (100 U/mL), and streptomycin (100 μg/mL) at 37 °C in a humidified atmosphere containing 5% CO_2_ (18). The aMPV subtype B strain Ck/B/Kaz/256/25 from the institute's collection was used in the study. Morphology was assessed by transmission electron microscopy (TEM) following negative staining (19).

RT-qPCR-positive samples were inoculated onto confluent Vero E6 monolayers and serially passaged until characteristic cytopathic effects (CPE) were observed. For virus propagation, infected cells were maintained in DMEM containing 1% FBS and antibiotics.

Following three freeze–thaw cycles, infected cultures were clarified by centrifugation at 1000 × g for 5 min. Virus-containing supernatants were aliquoted and stored at −70 °C.

Viral infectivity titers were determined in Vero E6 cells using the TCID_50_ method and calculated according to the Reed–Muench method (20). The fourth virus passage was used for challenge experiments, whereas the eleventh passage was utilized for vaccine preparation.

#### Concentration of viral particles

Virus-containing supernatants were concentrated by ultracentrifugation through a sucrose cushion (21). Briefly, clarified suspensions were layered onto 20% (w/v) sucrose prepared in phosphate-buffered saline (PBS, pH 7.4) and centrifuged at 100,000 × g for 2 h at 4 °C using a Beckman Coulter Optima ultracentrifuge equipped with an SW32Ti rotor.

Following centrifugation, supernatants were discarded, and viral pellets were resuspended in sterile PBS and stored at −80 °C until use. The morphology of the viral particles was evaluated by transmission electron microscopy (TEM) after negative staining.

#### Isolation of viral glycoproteins

Viral glycoproteins were isolated from concentrated viral particles using a detergent-based solubilization method. Briefly, purified viral suspensions were incubated with 4% (w/v) MEGA-10 detergent in PBS (pH 7.4) under gentle agitation at 4 °C for 30–60 min to disrupt the viral envelope and release surface glycoproteins while preserving their antigenic structure (22).

After solubilization, insoluble viral material was removed by centrifugation at 10,000–15,000 × g for 20–30 min at 4 °C. Supernatants containing solubilized glycoproteins were dialyzed extensively against PBS with repeated buffer replacement to remove residual detergent.

The resulting glycoprotein preparations were concentrated using ultrafiltration membranes and sterilized through 0.22 μm membrane filters. Protein concentration was determined using the Bradford assay according to the manufacturer's instructions.

#### SDS–PAGE and western blot analysis

Protein composition and integrity of concentrated viral preparations and purified glycoproteins were evaluated by SDS–PAGE and Western blot analysis (23).

Samples were mixed with Laemmli sample buffer containing 5% β-mercaptoethanol and heated at 95 °C for 5 min before electrophoresis in 10% polyacrylamide gels under reducing conditions. Gels were stained with Coomassie Brilliant Blue R-250 and destained in methanol/acetic acid solution. Protein molecular weights were estimated using prestained molecular weight markers (Thermo Fisher Scientific, USA).

For Western blot analysis, proteins were electrotransferred onto PVDF membranes using a semi-dry transfer system. Membranes were blocked with 5% skim milk in PBS containing 0.05% Tween-20 and incubated overnight at 4 °C with either anti- aMPV subtype B hyperimmune chicken sera diluted 1:1000.

Following washing, membranes were incubated with horseradish peroxidase-conjugated secondary antibodies for 1 h at room temperature. Protein bands were visualized using enhanced chemiluminescence substrate and documented using a ChemiDoc imaging system (Bio-Rad, USA).

#### Preparation of vaccine formulations

Purified viral glycoproteins were formulated with Montanide ISA 70 VG adjuvant (24). Briefly, 10 μg of glycoprotein antigen was emulsified with Montanide ISA 70 VG at a ratio of 30:70 (aqueous phase:oil phase) according to the manufacturer's recommendations. Homogenization was performed under continuous stirring until a stable water-in-oil emulsion was obtained.

Before administration, vaccine formulations were equilibrated at room temperature (20 °C−25 °C) for 6–9 h. Vaccine vials were mixed thoroughly immediately before and during use to maintain emulsion homogeneity.

Physical stability of the formulations was evaluated visually by monitoring emulsion uniformity and phase separation during storage at 4 °C.

Antigen association efficiency was determined by quantifying residual free antigen after centrifugation using a protein assay. Antigen association efficiency (%) = [(Total antigen – Free antigen)/Total antigen] × 100.

All vaccine formulations were prepared independently at least three times to ensure reproducibility.

Immunization.

Seven-day-old chickens were randomly assigned into experimental groups (Table 1). Birds received a single intramuscular immunization of 0.35 mL vaccine preparation containing glycoproteins formulated with Montanide ISA 70 VG or 0.03 mL live vaccine into one eye. Booster immunization with the same formulation was administered 3 weeks later. Control groups received phosphate-buffered saline (PBS).

**Table 1:** Experimental design of the vaccination study.

Vaccine	Number of birds	Age at primary vaccination	Vaccine dose	Route of administration	Booster immunization	Blood sampling	peripheral blood leukocytes sampling
PBS (Control)	15	7 days	350 μL	Intramuscular	Day 21	Days 7, 14, 21, 28, 56, 112, 168	Week 1, 2, 3, 4, 5, 6
HIPRAVIAR SHS	15	7 days	1 dose/bird	Ocular	None	Days 7, 14, 21, 28, 56, 112, 168	Week 1, 2, 3, 4, 5, 6
Experimental subunit vaccine	15	7 days	10 μg antigen in 350 μL	Intramuscular	Day 21	Days 7, 14, 21, 28, 56, 112, 168	Week 1, 2, 3, 4, 5, 6

#### ELISA and virus neutralization assays

Specific IgG antibodies against avian metapneumovirus (aMPV) subtype B were measured in serum samples using the IDEXX Avian Rhinotracheitis Antibody Test Kit (IDEXX Laboratories, Westbrook, ME, USA) according to the manufacturer's instructions. Serum samples were diluted 1:500, and antibody responses were expressed as sample-to-positive (S/P) ratios.

Virus-neutralizing antibody titers were determined using Vero E6 cells. Heat-inactivated serum samples were subjected to two-fold serial dilutions (ranging from 1:2 to 1:4096). Equal volumes of each serum dilution and a standardized suspension of aMPV subtype B containing 100 TCID_50_ were mixed and incubated for 60 min at 37 °C prior to inoculation onto confluent Vero E6 cell monolayers. Each serum dilution was tested in quadruplicate wells. The cultures were maintained at 37 °C in a humidified atmosphere containing 5% CO_2_ and monitored daily for cytopathic effects (CPE) for 7 days. Virus back-titration was performed in parallel to confirm the input dose of 100 TCID_50_. Neutralizing antibody titers were calculated using the Reed–Muench method.

#### Analysis of cytokine expression

Approximately 1–2 mL of peripheral blood was collected from the brachial (wing) vein of immunized chickens into sterile tubes containing sodium heparin as an anticoagulant. Peripheral blood mononuclear cells (PBMCs) were isolated by density-gradient centrifugation using Ficoll-Paque™ PLUS (Cytiva, Uppsala, Sweden). Whole blood was carefully layered onto Ficoll-Paque™ PLUS and centrifuged at 400 × g for 30 min at room temperature without brake. The PBMC layer was collected, washed twice with sterile phosphate-buffered saline (PBS), pelleted by centrifugation, and immediately processed for RNA extraction.

Total RNA was extracted from isolated PBMCs using TRIzol™ Reagent (Thermo Fisher Scientific, Waltham, MA, USA) according to the manufacturer's instructions. RNA concentration and purity were determined spectrophotometrically using a NanoDrop 2000 spectrophotometer (Thermo Fisher Scientific, Waltham, MA, USA). Only RNA samples with an A260/A280 ratio of 1.8–2.1 were used for subsequent analyses.

First-strand cDNA was synthesized from 1 μg of total RNA using the Vazyme Reverse Transcription Kit (Vazyme, Nanjing, China) according to the manufacturer's instructions.

Quantitative real-time PCR (RT-qPCR) was performed on an Applied Biosystems 7500 Fast Real-Time PCR System (Thermo Fisher Scientific, Waltham, MA, USA). Pre-designed TaqMan™ Gene Expression Assays (Thermo Fisher Scientific) were used for chicken IFN-γ (Assay ID: Gg03348618_m1) and IL-4 (Assay ID: Gg03370159_m1). Expression of 28S rRNA was quantified using a custom TaqMan assay with the following oligonucleotides: forward primer 5′-AGGACCGCTACGGACCTCC-3′, reverse primer 5′-CAACTGCATGATAGGAAGAGCCA-3′, and probe 5′-FAM-AGGACCGCTACGGACCTCCACCA-TAMRA-3′. Amplification reactions were carried out in a final volume of 20 μL containing TaqMan Universal PCR Master Mix, TaqMan Gene Expression Assays (or the custom TaqMan assay for 28S rRNA), and cDNA template. Thermal cycling conditions consisted of an initial activation step at 95 °C for 10 min, followed by 40 cycles of 95 °C for 15 s and 60 °C for 1 min.

Expression levels of IFN-γ and IL-4 were normalized to 28S ribosomal RNA (28S rRNA), which was used as the endogenous reference gene. Relative gene expression levels were calculated using the 2^−Δ*ΔCt*^ method, with samples from the non-immunized control group serving as the calibrator. All reactions were performed in triplicate, and no-template controls were included in each RT-qPCR run.

#### Sequencing and phylogenetic analysis

PCR products corresponding to the aMPV subtype B F gene were purified using the QIAquick PCR Purification Kit (Qiagen, Germany) and subjected to bidirectional Sanger sequencing.

Sequence chromatograms were edited and assembled using BioEdit software. Multiple sequence alignments were generated using ClustalW implemented in MEGA version 10.

Phylogenetic analysis of avian metapneumovirus based on the fusion (F) gene nucleotide sequence (1527 bp). The phylogenetic tree was constructed in Geneious using the Neighbor-Joining method with 1,000 bootstrap replicates. The Kazakhstan isolate is indicated in bold. Bootstrap values greater than 70% are shown at the branch nodes. Representative reference sequences of avian metapneumovirus subtypes B, including vaccine strains, were retrieved from GenBank and included in the analysis. Reference sequences representing avian metapneumovirus subtypes B were retrieved from GenBank for comparative analysis.

#### Bioinformatic and statistical analysis

Statistical analyses were performed using GraphPad Prism software version 8.0.2 (GraphPad Software Inc., USA). Data are presented as mean ± standard deviation (SD). Statistical analyses were performed using two-way analysis of variance (ANOVA) followed by Tukey's multiple comparisons test. A *P* value < 0.05 was considered statistically significant. Tukey's post hoc test was used to control for multiple pairwise comparisons.

## Results

## Detection and isolation of avian metapneumovirus

In the present study, samples collected from 16 commercial poultry farms located in three regions of Kazakhstan were investigated. A total of 115 chicken flocks, including broilers, broiler breeders, and layers, were examined for the presence of aMPV subtype B. Overall, 23 (20.0%) flocks tested positive for aMPV subtype B by real-time RT-PCR, with the majority of positive samples detected during the spring season. The distribution of tested flocks, RT-PCR-positive samples, and virus isolation results is summarized in Table 2.

**Table 2:** Detection of avian metapneumovirus in 115 chicken flocks from 16 commercial poultry farms in Kazakhstan (2024–2025).

Flock type	No. tested	Birds sampled	Pooled samples	RT-PCR positive	aMPV subtype	Virus isolates obtained
Broilers	15	90	45	2	B	0
Broiler breeders	87	535	268	20	B	0
Layers	13	65	32	1	B	1
Total	115	690	345	23	B	1

**Figure 1:**
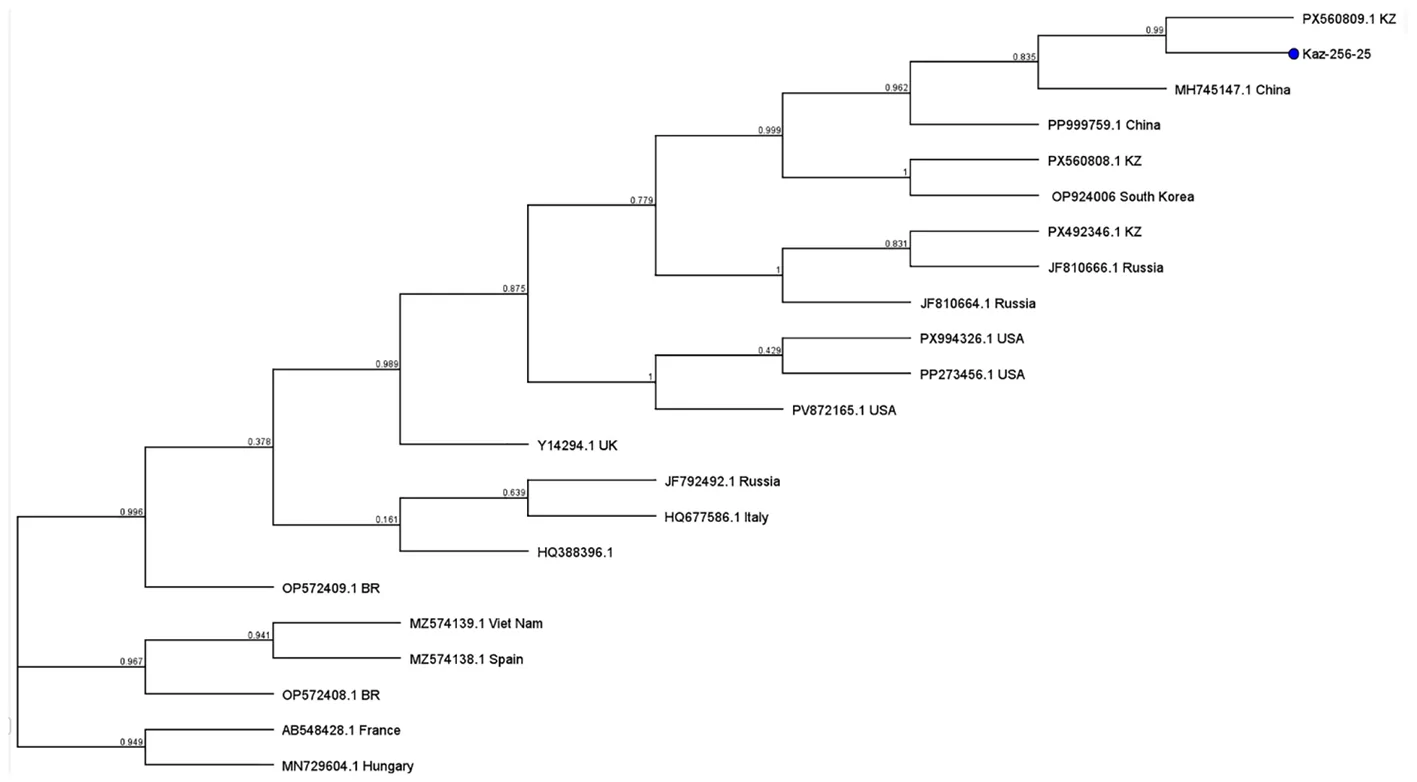
Phylogenetic analysis of avian metapneumovirus based on the fusion (F) gene nucleotide sequence. The phylogenetic tree was constructed in Geneious using the Neighbor-Joining method with 1,000 bootstrap replicates. The Kazakhstan isolate is indicated in bold. Bootstrap values greater than 70% are shown at the branch nodes. Representative reference sequences of avian metapneumovirus subtypes B, including vaccine strains, were retrieved from GenBank and included in the analysis.

Sequence analysis demonstrated that all detected viruses belonged to avian metapneumovirus subtype B (aMPV-B). Phylogenetic analysis further showed that the Kazakhstan isolates clustered within subtype B and were closely related to vaccine-like strains while forming a distinct monophyletic lineage, suggesting regional genetic divergence (Figure 1).

To isolate the virus, aMPV subtype B-positive field samples that tested negative for infectious bronchitis virus (IBV), Newcastle disease virus (NDV), infectious laryngotracheitis virus (ILTV), and *Mycoplasma gallisepticum* were selected for propagation in Vero cells. Following each passage, infected cell monolayers were treated with trypsin and subcultured into fresh culture flasks. Cytopathic effects (CPE), characterized by cell rounding, detachment, and floating cells, became evident after the third to fourth passages. Although 23 samples were positive for aMPV subtype B by multiplex real-time RT-PCR, infectious virus could not be maintained during serial passage in all but one sample. Consequently, only a single isolate was successfully propagated in Vero cells.

**Figure 2:**
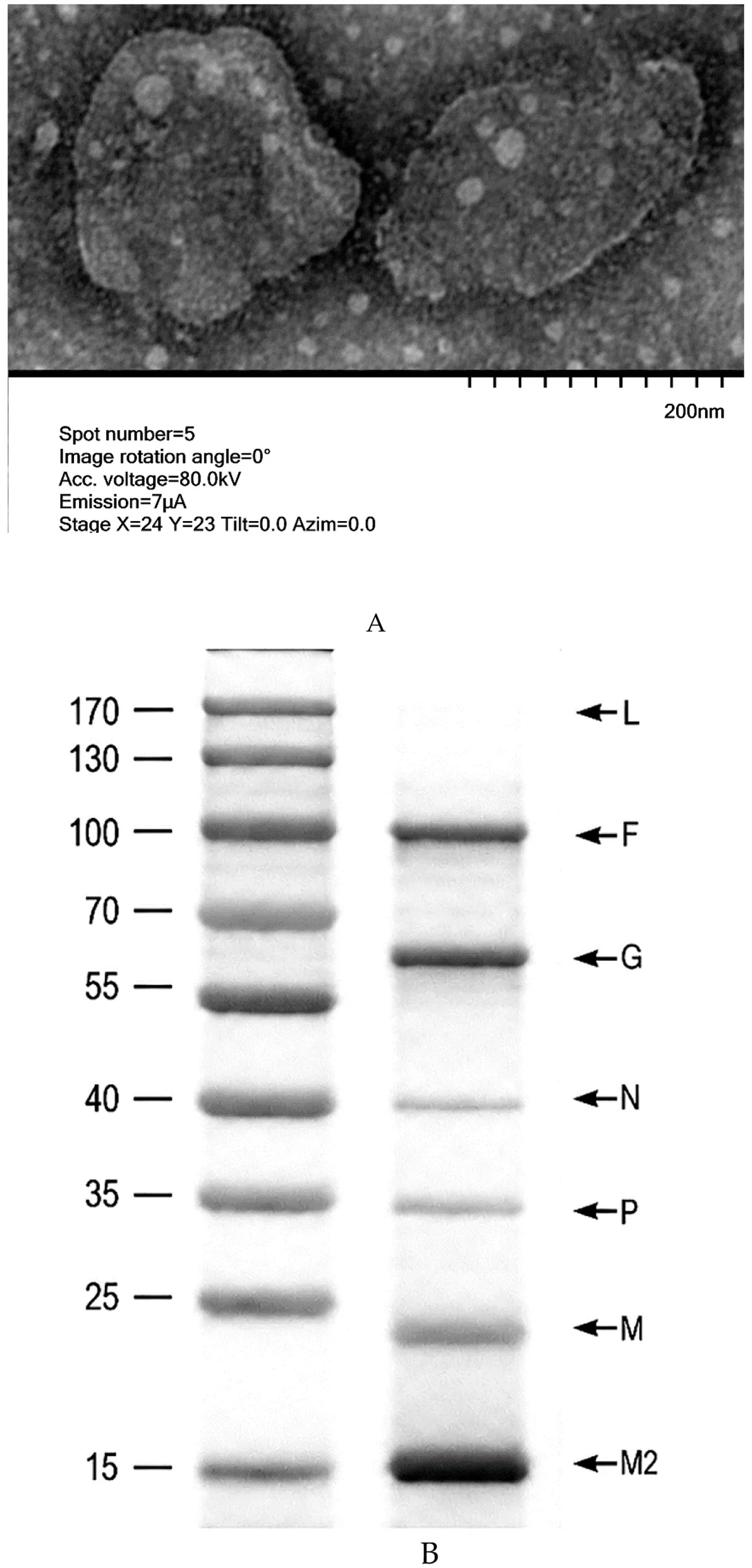
Characterization of the avian metapneumovirus isolate Ck/B/Kaz/256/25. **(A)** Transmission electron microscopy (TEM) of purified virus particles following negative staining, showing the characteristic morphology of enveloped avian metapneumovirus virions. Scale bar = [200 nm]. **(B)** SDS-PAGE analysis of purified viral proteins. Molecular weight markers (kDa) are indicated on the left.

The isolate, designated Ck/B/Kaz/256/25, was recovered from a laying hen. Negative-staining transmission electron microscopy revealed enveloped viral particles with morphology consistent with avian metapneumovirus (Figure 2). The identity of the isolate was further confirmed by electrophoretic analysis. This isolate was subsequently selected as the source of antigen for the development of the experimental subunit vaccine.

## Preparation of the subunit vaccine against avian metapneumovirus

**Figure 3:**
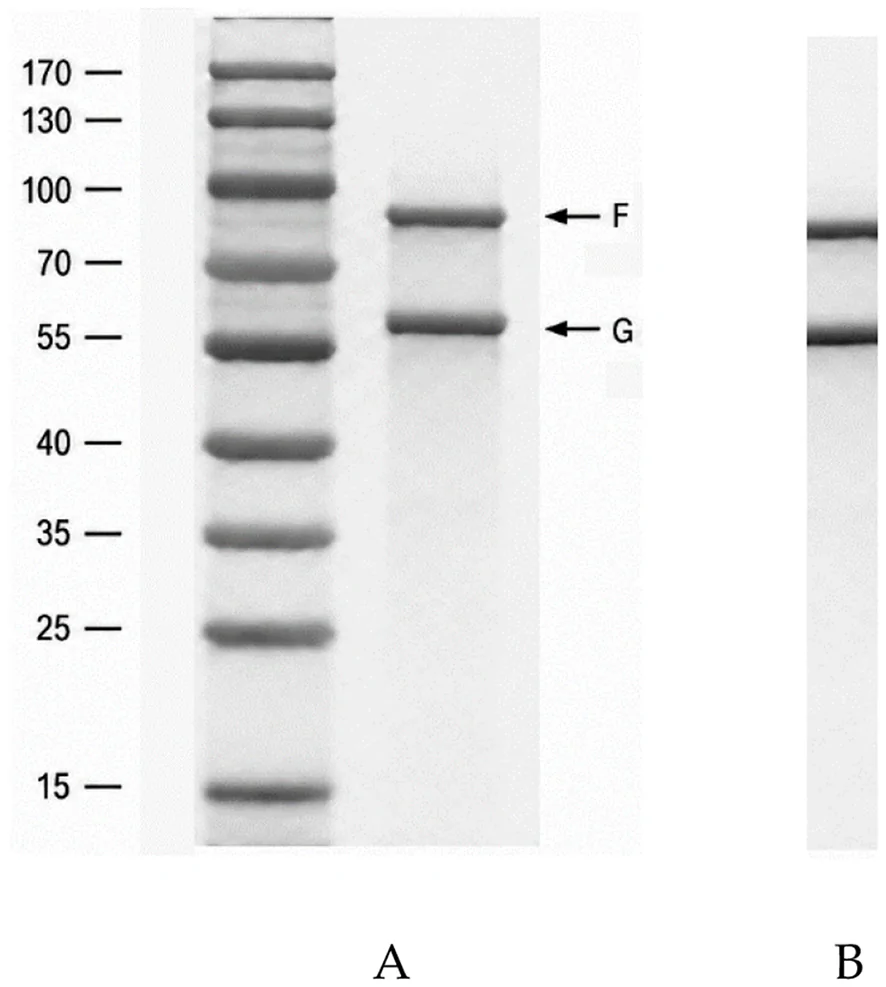
Characterization of the glycoprotein fraction obtained from the avian metapneumovirus isolate Ck/B/Kaz/256/25. **(A)** SDS-PAGE analysis of the detergent-solubilized viral glycoprotein fraction. Molecular weight markers (kDa) are indicated on the left. **(B)** Immunoblot analysis of the glycoprotein fraction using chicken anti-aMPV hyperimmune serum, confirming the antigenicity of the viral glycoproteins. The positions of the immunoreactive protein bands are indicated.

Viral glycoproteins of the aMPV subtype B strain Ck/B/Kaz/256/25 were obtained by detergent treatment using MEGA-10. The purity of the glycoprotein preparation was confirmed by electrophoresis and immunoblotting analysis (Figure 3).

The purified glycoprotein preparation containing 10 μg of protein in a final volume of 100 μL was sterilized by filtration through a 0.22 μm membrane filter and subsequently emulsified with Montanide ISA 70 VG (Seppic, France) at an aqueous phase-to-oil phase ratio of 30:70, yielding a stable water-in-oil emulsion. The final vaccine formulation was characterized with respect to its antigen content, emulsion properties, sterility, and physicochemical stability. The characteristics of the developed subunit vaccine formulation are summarized in Table 3.

**Table 3:** Characterization of the developed subunit vaccine formulation.

Characteristic	Specification	Observation
Antigen	Purified aMPV subtype B glycoproteins	Confirmed
Antigen content	10 μg/dose	Standardized
Adjuvant	Montanide ISA 70 VG	Standardized
Emulsion type	Water-in-oil	Homogeneous
Antigen:adjuvant ratio	30:70	Standardized
Antigen binding efficiency	94.0 ± 0.2	Standardized
Sterility	0.22 μm filtration	Passed
Storage	4 °C	Stable during study
Visible phase separation	None	Not observed
Sedimentation	None	Not observed
Number of independently prepared batches	≥3	Comparable appearance

The final vaccine formulation was administered intramuscularly at a dose volume of 350 μL per bird.

## Serum IgG antibody levels

Seven-day-old chickens were immunized twice at a 3-week interval with either a live vaccine or a subunit vaccine (*n* = 15 per group). Specific antibodies against aMPV subtype B were evaluated in 15 chickens from each group at 7, 14, 21, 28, 56, 112, and 168 days after primary immunization using a commercial avian rhinotracheitis antibody ELISA kit (IDEXX, Maine, USA).

**Figure 4:**
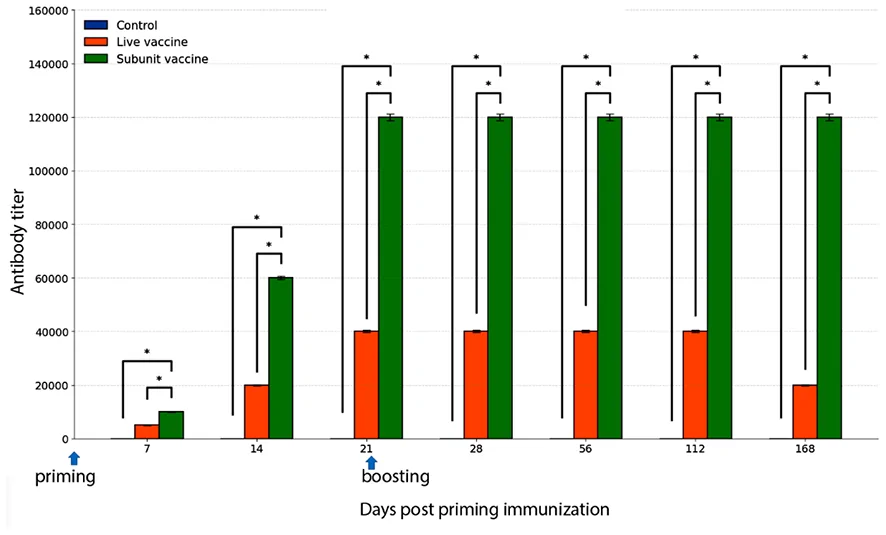
aMPV subtype B-specific IgG antibody levels in vaccinated chickens following primary and booster immunization with two vaccine formulations. Antibody responses were determined by indirect ELISA at the indicated time points after vaccination. Data are presented as mean ± SD (*n* = 15 birds per group). Statistical analysis was performed using two-way ANOVA followed by Tukey's multiple comparisons test using GraphPad Prism. *P* < 0.05 was considered statistically significant. Asterisks indicate statistically significant differences between the compared groups (*P* < 0.05).

As shown in Figure 4, the seroconversion rate in the subunit vaccine group reached 100% by day 14 after primary immunization. In contrast, the seroconversion rate in the live vaccine group was 60% and 100% on days 14 and 21 post-immunization, respectively.

At 21 days after primary vaccination, the mean antibody titer in the subunit vaccine group reached 120,000, whereas the antibody level in the live vaccine group did not exceed 40,000. Following booster immunization, no substantial increase in antibody titers was observed. However, by day 168 after the first immunization, antibody levels in the live vaccine group had declined to 20,000, while high humoral immune responses were maintained throughout the observation period in the subunit vaccine group.

These results indicate that the subunit vaccine exhibited higher antigenicity, characterized by earlier seroconversion, as well as stronger immunogenicity, resulting in higher and longer-lasting antibody responses compared with the live vaccine.

## Virus neutralizing antibody levels

The induction of virus-neutralizing (VN) antibodies following vaccination is considered an important indicator of protective immune responses. We evaluated VN antibody levels in 15 chickens from each group after immunization with either the live or the subunit vaccine.

**Figure 5:**
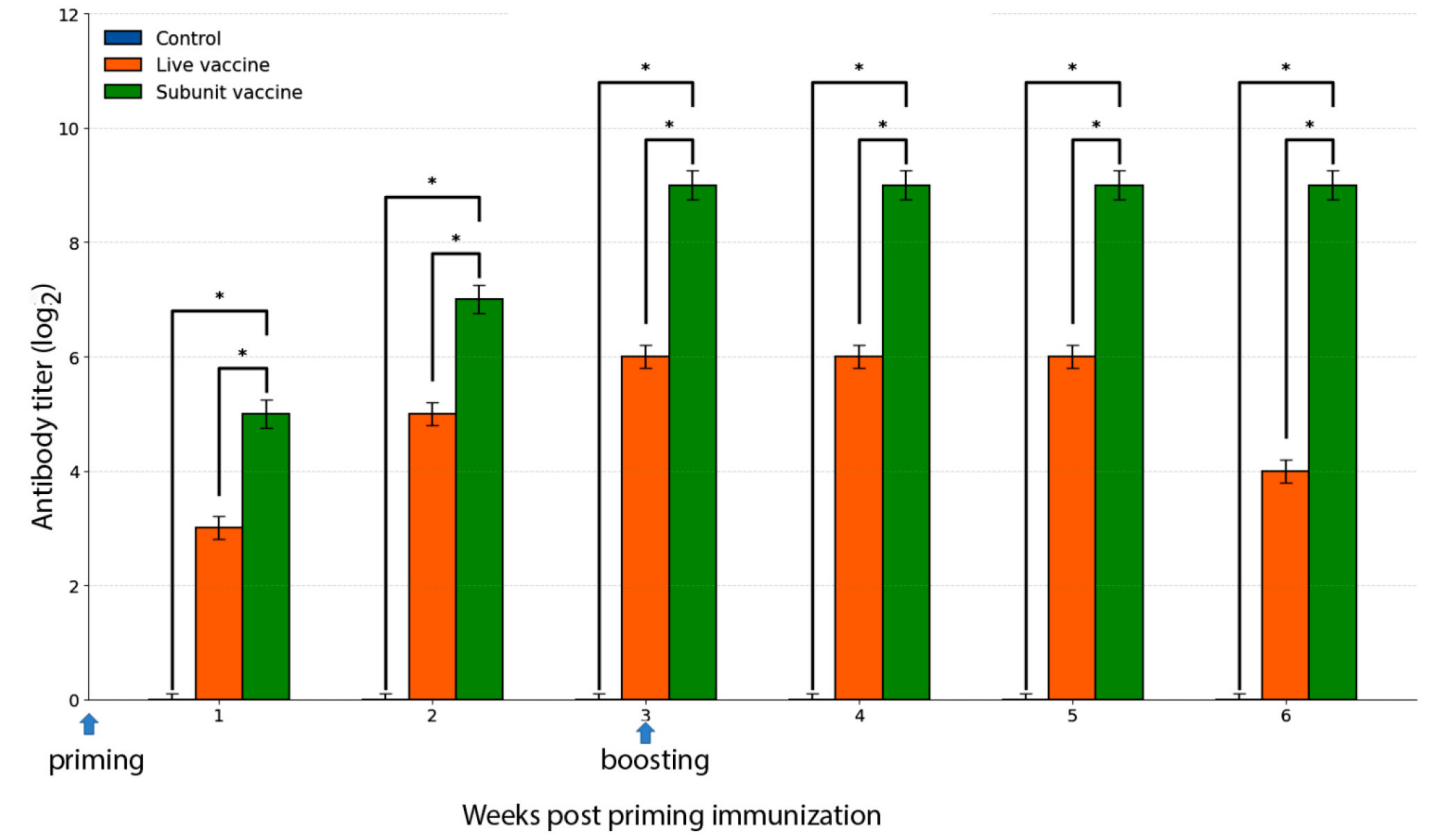
Virus-neutralizing antibody titers in vaccinated chickens following primary and booster immunization. Neutralizing antibody titers were determined at the indicated time points after vaccination. Data are presented as mean ± SD (*n* = 15 birds per group). Statistical analysis was performed using two-way ANOVA followed by Tukey's multiple comparisons test using GraphPad Prism. *P* < 0.05 was considered statistically significant. Asterisks indicate statistically significant differences between the compared groups (*P* < 0.05).

In both vaccinated groups, VN antibodies were detected as early as 7 days after vaccination, while a 100% positive response was observed by day 21 post-vaccination. Three weeks after primary and booster immunization, the geometric mean VN antibody titers in the live vaccine group were 6.0 log_2_ and 4.2 log_2_, respectively, whereas in the subunit vaccine group the corresponding titers reached 9.0 log_2_ and 9.2 log_2_ (Figure 5).

Thus, the VN antibody titer in the subunit vaccine group was approximately 8-fold higher than that in the live vaccine group after a single immunization and 16-fold higher after booster immunization (Figure 5). In contrast, VN antibodies in the non-vaccinated control group remained negative (< 2) throughout the study period.

## Expression of Th1- and Th2-associated cytokines in peripheral blood mononuclear cells

The expression of Th1- and Th2-associated cytokine mRNAs is considered a sensitive marker of lymphocyte activation *in vivo*. In this study, the mRNA expression levels of Th1- and Th2-associated cytokines, namely IFN-γ and IL-4, in PBMC samples collected 1–6 weeks after primary immunization with different vaccines were evaluated using quantitative real-time PCR (qRT-PCR). Relative gene expression levels compared with the control group were calculated using 28S rRNA as the reference gene.

**Figure 6:**
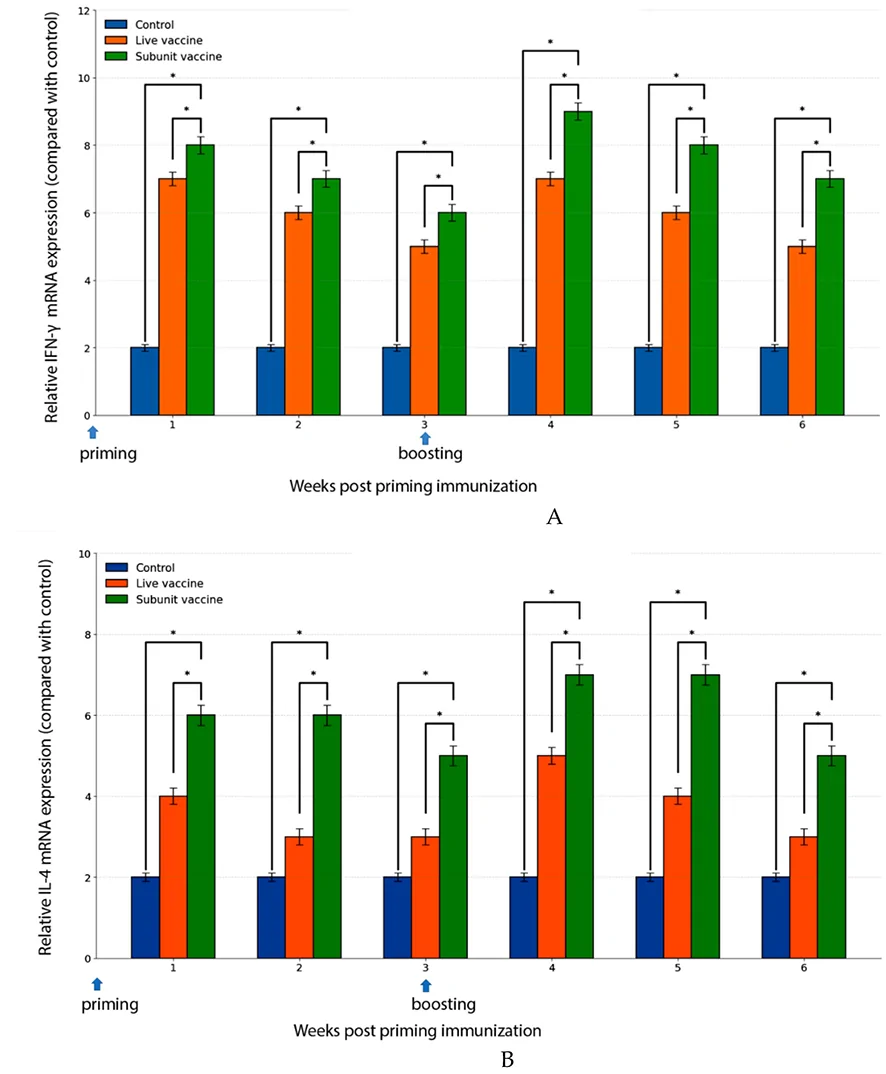
Cytokine mRNA expression in vaccinated chickens following primary and booster immunization. **(A)** IFN-γ and **(B)** IL-4 expression levels were determined by quantitative real-time RT-PCR in peripheral blood leukocytes at the indicated time points. Data are presented as mean ± SD (*n* = 15 birds per group). Statistical analysis was performed using two-way ANOVA followed by Tukey's multiple comparisons test in GraphPad Prism. *P* < 0.05 was considered statistically significant; Asterisks indicate statistically significant differences between the compared groups (*P* < 0.05).

The mRNA expression level of IFN-γ in the subunit vaccine group was approximately 16-fold higher than that in the control group and 4-fold higher than that observed in the live vaccine group. Moreover, booster immunization further increased IFN-γ expression levels by approximately 2-fold (Figure 6A). A similar pattern was observed for IL-4 expression (Figure 6B).

These results demonstrated that the subunit vaccine effectively stimulated the expression of both IL-4 and IFN-γ following immunization and induced stronger cytokine responses in peripheral blood leukocytes compared with the live vaccine.

## Discussion

Vaccination against avian metapneumovirus (aMPV) infection is an important component of respiratory disease prevention in commercial and breeding poultry production. The virus can cause respiratory tract disorders, reduced productivity, deterioration in egg quality, and increased susceptibility to secondary infections, resulting in significant economic losses. The use of vaccines helps to develop specific immunity, reduce clinical manifestations of the disease, and limit viral spread within a flock. Currently, different types of vaccines are available for the prevention of aMPV infection, including live attenuated and inactivated vaccines. Live vaccines provide rapid induction of both local and systemic immunity, whereas inactivated vaccines are mainly used to enhance and prolong protection in replacement pullets and adult birds. The choice of vaccination strategy and vaccine type depends on the epidemiological situation, poultry production system, and management practices (25–28).

The obtained results demonstrated that the developed subunit vaccine against avian metapneumovirus exhibited pronounced immunogenic activity and induced a stronger and more sustained immune response compared with the live vaccine. This was confirmed by the levels of humoral immunity, virus-neutralizing antibodies, and the expression of Th1- and Th2-associated cytokines.

One of the key indicators of vaccine efficacy is the rate of seroconversion and the level of specific antibodies (29). In the present study, the subunit vaccine induced 100% seroconversion by day 14 after primary immunization, whereas the same level was achieved only by day 21 in the live vaccine group. Earlier seroconversion indicates high antigenic activity of the subunit formulation and its ability to rapidly induce adaptive immune responses. Moreover, IgG antibody levels in the subunit vaccine group were substantially higher than those observed in the live vaccine group throughout the entire observation period. The persistence of high antibody titers up to 168 days after vaccination suggests the development of long-lasting humoral immunity, which is particularly important in commercial poultry production, where the duration of protection directly affects the epizootic status of poultry farms.

Virus-neutralizing (VN) antibodies are another important criterion for evaluating vaccine protective potential, since they are directly involved in preventing viral entry into target cells (30). In the present study, the subunit vaccine induced significantly higher VN antibody levels compared with the live vaccine. Three weeks after primary immunization, VN antibody titers were approximately 8-fold higher, while after booster immunization they were 16-fold higher than those induced by the live vaccine. These findings suggest that the subunit vaccine construct effectively preserved the immunodominant epitopes of the viral antigen required for the induction of functionally active humoral immunity. In addition, the absence of detectable VN antibodies in the control group confirmed the specificity of the induced immune response.

Analysis of the cytokine profile demonstrated that the subunit vaccine stimulated both cellular and humoral immune responses (31). The significant increase in IFN-γ expression indicates activation of Th1-mediated immunity, which plays a critical role in antiviral defense and elimination of infected cells. Simultaneously, elevated IL-4 expression reflected activation of the Th2 response associated with B-cell differentiation and antibody production. Therefore, the investigated vaccine induced a balanced Th1/Th2 immune response, which represents an important advantage in the development of modern subunit vaccines. The higher expression levels of both cytokines compared with the live vaccine may be associated with optimized antigen presentation and enhanced immune stimulation provided by the vaccine platform.

The obtained data are consistent with current concepts regarding the advantages of subunit vaccines, including their high safety profile, absence of reversion-to-virulence risk, and the possibility of targeted immune stimulation. Unlike live vaccines, whose efficacy may be reduced by maternal antibodies or the circulation of field viral variants, subunit vaccines allow more flexible adaptation of antigen composition and provide more stable immune responses.

## Study limitations

The present study has several limitations that should be considered when interpreting the findings. First, although the vaccine formulation induced robust humoral and cellular immune responses, its protective efficacy was not evaluated in a homologous or heterologous challenge model. Second, the protective performance of the vaccine under commercial poultry production conditions was not assessed and should be investigated in future field studies. Third, the evaluation of mucosal immunity was limited to the analysis performed in this study and did not include additional markers such as secretory IgA or mucosal cellular immune responses. Furthermore, the vaccine was developed using a single avian metapneumovirus subtype B isolate; therefore, its cross-protective potential against genetically diverse aMPV strains remains to be determined. Although the physicochemical properties of the nanocomplexes were characterized, additional studies evaluating long-term stability, batch-to-batch reproducibility, and other formulation quality attributes would further strengthen the characterization of the vaccine. Finally, the relatively small number of animals included in each experimental group may have limited the statistical power for detecting subtle differences between experimental groups. These limitations should be addressed in future studies to further validate the efficacy and broader applicability of the proposed vaccine platform.

Taken together, the results of this study indicate the high potential of the developed subunit vaccine against aMPV subtype B as a promising tool for the specific prevention of avian metapneumovirus infection. The high levels of specific and virus-neutralizing antibodies, together with the pronounced induction of IFN-γ and IL-4 cytokines, confirm the ability of the vaccine to induce long-lasting and comprehensive immune responses. These findings provide a basis for further studies aimed at evaluating the protective efficacy of the vaccine under experimental challenge and commercial field conditions.

## Conclusion

The present study demonstrated that the developed subunit vaccine against aMPV subtype B induced strong and durable immune responses in vaccinated chickens and outperformed the live vaccine in several key immunological parameters. The subunit vaccine promoted earlier seroconversion, generated significantly higher levels of specific IgG and virus-neutralizing antibodies, and maintained elevated antibody titers over an extended period following immunization.

In addition to enhanced humoral immunity, the vaccine stimulated both Th1- and Th2-associated immune responses, as evidenced by the increased expression of IFN-γ and IL-4 cytokines. The simultaneous activation of cellular and humoral immunity suggests the establishment of a balanced and comprehensive immune response, which is essential for effective antiviral protection.

Overall, the findings highlight the strong immunogenic potential of the developed subunit vaccine and support its consideration as a promising candidate for the prevention of aMPV subtype B infection in poultry. Further investigations involving experimental challenge studies and field evaluation are warranted to confirm its protective efficacy and practical applicability under commercial poultry production conditions.

## Data Availability

The original contributions presented in the study are included in the article/supplementary material, further inquiries can be directed to the corresponding author/s.
